# TIKI2 is upregulated and plays an oncogenic role in renal cell carcinoma

**DOI:** 10.18632/oncotarget.7873

**Published:** 2016-03-03

**Authors:** Xiaodong Yuan, Baijun Dong, Yunze Xu, Liang Dong, Jiwei Huang, Jin Zhang, Yonghui Chen, Wei Xue, Yiran Huang

**Affiliations:** ^1^ Department of Urology, Ren Ji Hospital, School of Medicine, Shanghai Jiao Tong University, Shanghai 200127, China

**Keywords:** TIKI2, renal cell carcinoma, Wnt antagonist, β-catenin, Wnt

## Abstract

TIKI2 is a negative regulator of the Wnt family. Although many Wnt antagonists play important roles in renal cell carcinoma (RCC), the molecular function of TIKI2 in human RCC has not been fully elucidated. Here, we analyzed TIKI2 mRNA level in RCC specimens, the corresponding non-tumor tissues, RCC cell lines, and human proximal tubule epithelial cell line HK-2 using qPCR. We demonstrated that TIKI2 was highly expressed in RCC tissue (*P* < 0.05) and most RCC cell lines. *In vitro*, TIKI2 knockdown significantly inhibited proliferation, invasion, and clone formation ability of 769-P cells compared with controls, while ectopic TIKI2 expression enhanced A498 cell proliferation, invasion, and clone formation ability. *In vivo*, the average tumor volume was significantly increased in mice injected with A498-Tiki2 cells (*P* < 0.05). In the 769-P cell TIKI2 knockdown group, the average tumor volume was not significantly different compared to that of the control group (*P* = 0.08). Moreover, Wnt/β-catenin signaling was not affected by TIKI2 knockdown or overexpression. Results of the present study indicate that TIKI2 is upregulated in RCC tissues and plays an oncogenic role in RCC.

## INTRODUCTION

Approximately 61,560 Americans are expected to be diagnosed with cancer of the kidney and renal pelvis in 2015, and 14,080 deaths are predicted to be related to this disease [1]. Although targeted therapies such as vascular endothelial growth factor and mammalian target of rapamycin inhibitors have transformed the management of metastatic renal cell carcinoma (RCC), these drugs have shown limited efficacy and metastatic RCC remains incurable [2]. Therefore, there has been much interest in investigating the other important signaling pathways involved in the pathogenesis of RCC.

Wnt signaling is tightly regulated during kidney development and plays an important role in a variety of kidney diseases including RCC [3-8]. In the activated canonical Wnt pathway, β-catenin accumulates in the cytoplasm and translocates to the nucleus, where it induces transcription of T-cell transcription factor/lymphocyte enhancer factor target genes [9]. There are two non-canonical Wnt pathways, Wnt-calcium pathway, and the planar cell polarity (PCP) pathway, in which β-catenin is not involved. Along with the above, Wnt signaling also regulates some other pathways that have not yet been extensively elucidated.

To date, many Wnt antagonists have been found, such as the secreted Frizzled-related proteins (sFRP), Wnt inhibitory factor1 (WIF-1), Cerberus and the Dickkopf (DKK) family proteins. Most Wnt antagonists have been shown to contribute to the development of RCC [7]. Zhang *et al*. recently identified a new Wnt antagonist named TIKI (also known as TRABD2B) that dampens Wnt signaling by removing several amino-terminal residues from Wnt proteins [10]. TIKI consists of three domains: an amino-terminal signal peptide; an ectodomain, also referred to as the TIKI domain; and a transmembrane domain. In human, there are two TIKI orthologs, TIKI1 and TIKI2. Li *et al*. reported that TIKI2 can suppress the growth of osteosarcoma by inactivating Wnt/β-catenin signaling [11].

We analyzed the Oncomine database to gain insight into the expression alteration of TIKI2 in RCC and observed that TIKI2 was highly expressed in RCC. We then investigated *TIKI2* expression in RCC specimens and cell lines and found that *TIKI2* was upregulated in RCC and TIKI2 was able to promote RCC growth. Our results suggest that TIKI2 may be a promising target for RCC.

## RESULTS

### TIKI2 was highly expressed in RCC specimens

**Figure 1:**
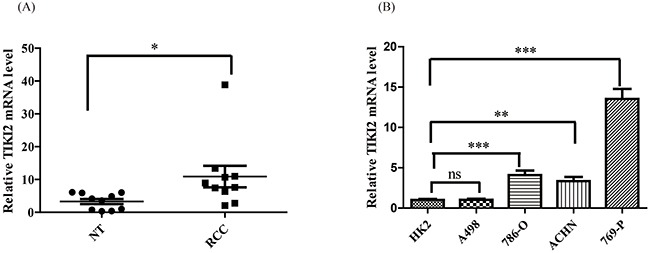
TIKI2 was highly expressed in RCC specimens and cell lines **A.** TIKI2 mRNA level in RCC specimens and the corresponding non-tumor tissues were obtained using quantitative real-time PCR. Higher TIKI2 mRNA level was observed in RCC specimens than in the corresponding non-tumor tissues (*n* = 10, data are mean ± SEM). **B.** TIKI2 mRNA expressions of four RCC cell lines (A498, 786-O, 769-P, and ACHN) and one normal human proximal tubule epithelial cell line HK-2 were determined using quantitative real-time PCR. Higher TIKI2 mRNA level was also observed in most RCC cell lines (786-O, ACHN, 769-P) compared with that in HK-2 cells. No significant difference in TIKI2 mRNA level was observed between A498 and HK-2 cells. *P < 0.05, **P < 0.01, ***P < 0.001; ns: not significant; NT: corresponding non-tumor tissues.

To determine TIKI2 expression in RCC, we analyzed the Oncomine database and found that TIKI2 was upregulated in RCC compared with normal kidney tissue (Supplementary Figure S1) [12]. We then examined TIKI2 mRNA expression in our clinical RCC specimens using qPCR. TIKI2 was dramatically upregulated in RCC samples (*n* = 10) compared to that in the corresponding non-tumor tissues (Figure 1A and Supplementary Figure S2). Meanwhile, TIKI2 mRNA was also significantly increased in most RCC cell lines compared with HK-2 cells (Figure 1B).

### TIKI2 promotes RCC proliferation, invasiveness, and colony formation abilities

**Figure 2:**
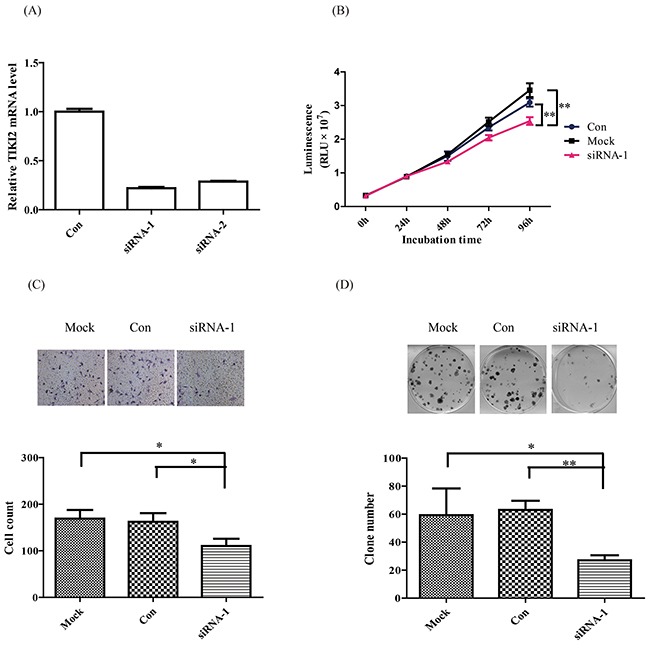
TIKI2 knockdown suppressed RCC cell proliferation, invasiveness, and colony formation abilities TIKI2-related loss of function was achieved by TIKI2 siRNA knockdown in 769-P cells, which had the highest endogenous TIKI2 expression among the four RCC cell lines. **A.** TIKI2 expression was quantified by real-time PCR after 48-h siRNA transfection. TIKI2 mRNA levels in the siRNA-1 and siRNA-2 groups were decreased to about 30% of the endogenous TIKI2 expression in 769-P cells. **B.** Cell viability was determined using the CellTiter-Glo luminescent cell viability assay. TIKI2 knockdown inhibited the proliferation of 769-P cells compared with controls. **C.** TIKI2 knockdown decreased the invasiveness ability of 769-P cells compared with control; representative images are shown, original magnification, × 200. **D.** TIKI2 knockdown suppressed the colony formation ability of 769-P cells compared with control; representative images are shown. The data shown are mean ± SD of three replicates. *P < 0.05, **P < 0.01. Con: control; RLU: relative light unit.

Since TIKI2 was upregulated in RCC specimens and cell lines, we next investigated the role of TIKI2 on RCC cell behaviors. First, we checked the effect of *TIKI2* knockdown in 769-P cells that expressed the highest TIKI2 level among the RCC cell lines. We knocked down TIKI2 by using siRNA and confirmed the knockdown using qPCR (Figure 2A). After TIKI2 knockdown, cell proliferation was significantly suppressed compared with that of cells transfected with negative control (Figure 2B). TIKI2 knockdown also caused a significant decrease in the invasion capability of 769-P cells compared to negative control (Figure 2C). Moreover, the colony formation ability of TIKI2 knockdown 769-P cells was significantly decreased compared with that of the negative control (Figure 2D).

**Figure 3:**
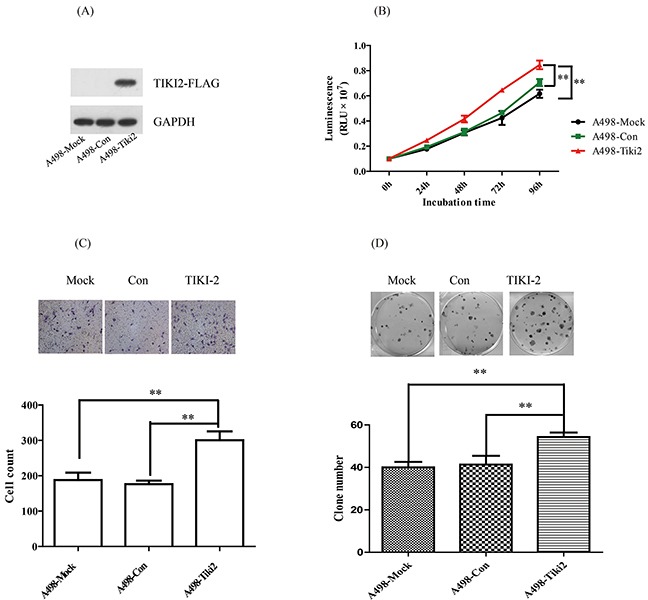
Ectopic TIKI2 expression promoted the proliferation, invasiveness, and colony formation abilities of RCC cells TIKI2-related gain of function was achieved by ectopic TIKI2 expression in stable A498 cells, which had the lowest endogenous TIKI2 mRNA level among the four RCC cell lines. **A.** Expression of ectopic TIKI2 in A498 cells was confirmed by Western blotting using an antibody against FLAG. Since a TIKI2 antibody is not commercially available, ectopic TIKI2 expression was labeled with FLAG. **B.** TIKI2 overexpression in A498 cells promoted cell proliferation compared with controls. **C.** TIKI2 overexpression increased the invasion capability of A498 cells compared with controls; representative images are shown, original magnification, × 200. **D.** TIKI2 overexpression increased the colony formation ability of A498 cells compared with controls; representative images are shown. The data are shown as mean ± standard deviation of three replicates. **P < 0.01. Con: control.

To further confirm the role of TIKI2 in the RCC cell lines, we constructed stable TIKI2 overexpressing A498 cell lines, which characteristically express the lowest TIKI2 mRNA level among the RCC cell lines, and confirmed their activity using western blotting (Figure 3A). Proliferation assays showed that the ectopic expression of TIKI2 in A498 cells dramatically promoted cell growth compared to the control cells (Figure 3B). TIKI2 overexpression in A498 cells also significantly increased their invasion capability compared to that of the control cells (Figure 3C). In addition, the colony formation ability of stable A498 TIKI2-expressing cells was significantly increased compared to that of control cells (Figure 3D).

### TIKI2 promotes RCC xenograft growth in mice

**Figure 4:**
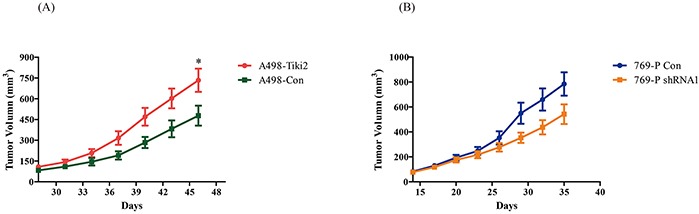
Effects of TIKI2 on human RCC xenografts **A.** Ectopic TIKI2 expression promoted A498 RCC xenograft growth. Xenograft tumors were established via the subcutaneous injection of 5 × 10^6^ A498-Tiki2 and A498-Con cells in the right flank area of nude mice (*n* = 5 per group, *P < 0.05 at the end of the observation period). **B.** TIKI2 knockdown suppressed 769-P RCC xenograft growth, although the difference was not significant. The 769-P shRNA1 and control cells (1 × 10^7^ each) were injected into the right flank area of nude mice (*n* = 5 per group, *P* = 0.08 at the end of the observation period). The tumor size was plotted as mean ± SEM.

To investigate the effect of TIKI2 *in vivo*, stable A498 cell lines ectopically expressing TIKI2 or controls were used on nude mice. The average tumor volume was significantly increased in mice injected with A498-TIKI2 cells (Figure 4A). We also injected 769-P stable cell lines infected with TIKI2-shRNA1 and control-shRNA into nude mice. The average tumor volume was decreased in the TIKI2-shRNA1 group compared with the control group, although the difference was not significant (Figure 4B; *P* = 0.08 at the end of the observation period). These data showed that TIKI2 could promote RCC xenograft growth in mice.

### TIKI2 does not affect the Wnt/β-catenin pathway in RCC cells

Since TIKI2 was reported to suppress the Wnt/β-catenin pathway in osteosarcoma, we next investigated whether TIKI2 could affect the Wnt/β-catenin pathway in RCC cells. Immunoblot analysis revealed that the ectopic TIKI2 expression of A498 cells did not decrease β-catenin levels in RCC cells (Supplementary Figure S3). The same results were observed in TIKI2 knockdown 769-P cells. Therefore, TIKI2 might promote RCC growth through other mechanisms.

## DISCUSSION

TIKI2, along with its ortholog TIKI1, was recently identified as a new Wnt antagonist with a different mechanism from those previously discovered [10]. Many Wnt antagonists, such as sFRP, WIF-1, and the DKK family, play important roles in RCC.[13-19] Therefore, here we investigated the role of TIKI2 in RCC and discovered for the first time that TIKI2 is highly expressed in human RCC specimens and could promote RCC growth.

The precise pathway by which TIKI2 exerts its effect on RCC is unclear. First, as Zhang *et al*. demonstrated that TIKI2 can not only cleave the canonical Wnt-Wnt3a but also cleave the non-canonical Wnts such as Wnt5a [10], TIKI2 may cleave Wnt3a to inactivate the Wnt canonical pathway in RCC. Wnt/β-catenin signaling is activated in RCC. However, we did not observe that TIKI2 overexpression in A498 cells could activate or inhibit Wnt canonical signaling by testing β-catenin level, which demonstrated that TIKI2 may not influence the Wnt canonical pathway in RCC. Second, TIKI2 may antagonize Wnt5a to affect RCC cellular behavior. The role of Wnt5a in different cancers is context-dependent. Gujral *et al.* demonstrated that Wnt5a and its cognate receptor Fzd2 are overexpressed in late-stage breast, lung, colon and liver cancers [20]. Conversely, Borcherding and colleagues found that Wnt5a could inhibit the expansion of tumor-initiating cells in breast cancer [21]. In the renal cell carcinoma TCGA dataset, more than 80% of specimens had lost one or two Wnt5a alleles [21], which suggested that Wnt5a may play an important role in RCC. The ectopic expression of Wnt5a reportedly suppressed human RCC cellular growth [22]. Moreover, active non-canonical Wnt signaling could inhibit canonical Wnt signaling [19]. Therefore, TIKI2 might cleave Wnt5a and then inhibit the non-canonical Wnt pathway in RCC to suppress the active effect of Wnt3a on the canonical pathway. Third, Wnt7A and Wnt10A, two other Wnt family members, were reported to be tumor suppressor gene and oncogene in RCC, respectively [23, 24]. Since TIKI2 could inactivate all other Wnt family members except for Wnt11, TIKI2 might antagonize other Wnt family members and promote RCC cellular behavior. Finally, TIKI2 may also promote RCC growth through other pathways. Zhang reported that TIKI2, which was named Hkat (heart, kidney, adipose-enriched transmembrane protein) in his study, was involved in adipogenesis [25]. Conclusive evidence has suggested that obesity is associated with an increased risk of renal cancer [26]. Although adipose tissue has been indicated as an active endocrine organ that could participate in many aspects of the pathology of metabolic syndromes [27], the mechanisms of obesity in RCC remain elusive. TIKI2 might be potentially involved in RCC by regulating adipogenesis-associated transcription factors. Taken together, these findings show that additional studies are required to investigate the precise mechanism of TIKI2 involvement in RCC.

In addition to the unidentified mechanism underlying TIKI2 mRNA upregulation in RCC, our study had another limitation. We found that TIKI2 was highly expressed in RCC based on transcription level only; however, we could not investigate the impact of TIKI2 expression on translation level using Western blotting on clinical specimens or immunostaining on tissue sections because the TIKI2 antibody is not commercially available.

In the present study, as far as we known, we reported for the first time that TIKI2 is upregulated in RCC. TIKI2 knockdown or overexpression suppressed or promoted RCC cell growth *in vitro* and *in vivo*, respectively. However the precise mechanism by which TIKI2 is involved in RCC requires further investigation. These results shed light on the oncogenic role of TIKI2 in RCC cells and raise the intriguing possibility that TIKI2 may be a potential new target for RCC treatment.

## MATERIALS AND METHODS

### Clinical specimens

Human RCC specimens and their corresponding non-tumor tissues were obtained from Renji Hospital Tissue Bank. The study protocol and use of the clinical specimens was approved by the Institutional Ethics Committee. All patients signed a written informed consent form. The specimens were collected after surgical resection, immediately frozen, and stored in liquid nitrogen until use.

### Cell culture

All human RCC cell lines and the human proximal tubule epithelial cell line HK-2 were obtained from the Cell Bank of the Chinese Academy of Sciences (Shanghai, China). RCC cell lines 786-O and 769-P were maintained in RPMI-1640 media (Hyclone, USA). The ACHN and A498 cell lines were maintained in MEM media (Hyclone, USA). HK-2 cell lines were maintained in DMEM/F12 (Hyclone, USA). All media were supplemented with 10% fetal bovine serum (FBS, Gibco, USA), 100 U/ml penicillin, and 100 μg/ml streptomycin. The cells were cultured at 37°C in a humidified atmosphere with 5% CO_2_.

### Quantitative real-time polymerase chain reaction (PCR)

Total RNA was isolated from the clinical specimens and cell lines using the TRizol reagent (Invitrogen, USA) and then transcribed into cDNA using PrimeScript RT Reagent Kit (Takara, Japan). Real-time PCR was performed using SYBR^®^ Premix Ex Taq™ II kit (Takara, Japan) on a 7500 Real-Time PCR System (Applied Biosystems, USA). Changes in TIKI2 gene expression were calculated using the ΔΔCt method. The ACTB gene was used as endogenous control to normalize expression data. The following primers were used: TIKI2-forward primer, 5′-GACCTGCGTGCTGATC-3′; and TIKI2-reverse primer, 5′-TAAAAGAAGATGACAG-3′; ACTB-forward primer, 5′-TTCTACAATGAGCTGCGTG-3′; and ACTB-reverse primer, 5′-CTCAAACATGATCTGGGTC-3′ (GenePharma, China).

### Stable cell lines construction

The pLenti-CMV-FLAG-PGK-Puro-TIKI2 plasmid, which expresses TIKI2, and the control plasmid were purchased from Obio Technology Company (China). The A498 cells were infected with a lentivirus that expresses TIKI2 and selected in puromycin (Invitrogen, USA). The clones were confirmed by western blotting. Stable cell lines were maintained in MEM supplemented with 10% FBS, and 2 μg/ml puromycin. Similar procedures were performed to generate stable cell lines expressing TIKI2-shRNA in 769-P cells using shRNA-1 (5′-GCACCCGTGTCTACTTTGA-3′). TIKI2 knockdown was confirmed by real-time PCR.

### TIKI2 knockdown by small interfering RNA

The 769-P cells were transfected with *TIKI2* small interfering RNA (siRNA) and negative control using Lipofectamine 2000 (Invitrogen, USA) according to the manufacturer's protocol. The mRNA expression was checked by qRT-PCR 48 h post-transfection. TIKI2 siRNA-1 (5′-GCACCCGTGTCTACTTTGA-3′) and siRNA-2 (5′-GAGCTTTACTGGCGCTTGA-3′) (GenePharma, China) were used.

### Western blotting

The cells were lysed in RIPA buffer (Thermo, USA) supplemented with a cocktail of protease inhibitors. Protein concentrations were determined using a BSA kit (Thermo, USA). The samples were separated on 10% SDS-PAGE gels and then transferred onto a nitrocellulose membrane. After blocking with 5% (w/v) nonfat dry milk, the membranes were incubated with antibodies against FLAG (1:2000; Sigma, USA), β-catenin (1:5000; Abcam, UK), and β-actin (1:1000, Santa Cruz, USA) overnight at 4°C. After incubation with horseradish peroxidase-conjugated secondary antibodies (Santa Cruz, USA), immunoreactive bands were detected with a chemiluminescent substrate and exposed to X-ray film.

### Cell proliferation assay

For the stable transfection cell proliferation assay, A498, A498-Con, A498-Tiki2 cells were seeded at a density of 2 × 10^3^ per well in 96-well microplates and incubated for 96 h. Cell viability was determined every 24 h using a CellTiter-Glo luminescent cell viability assay kit (Promega, USA), and measured by Varioskan Flash microplate reader (Thermo, USA) according to the manufacturer's protocol. Each experiment was repeated independently at least three times in triplicate. For the cell proliferation assays with TIKI2 siRNA, the 769-P cells were seeded at a density of 2 × 10^3^ per well in 96-well microplates the day before the transfection. After the transient transfection, the cells were incubated for 96 h using the same method as described above.

### Transwell invasion assay

The invasive capacity of the cells was evaluated by a transwell assay in a 24-well plate (Corning, USA) according to the manufacturer's protocol. Briefly, the upper chambers were coated with Matrigel (BD Biosciences, USA). For the stable transfection, the cells were suspended in Opti-MEM (Invitrogen, USA) reduced serum medium and seeded in the upper chambers. 500 μl medium with 10% FBS was added to the lower chambers. The cells were then allowed to invade the Matrigel matrix for 24 h. Thereafter, the transmigrated cells were fixed and stained with crystal violet and counted in five randomly selected microscopic fields. All of the experiments were performed in triplicate. For the transient transfection, the cells were transfected with siRNA for 48 h and then suspended and seeded as described above.

### Clone formation assay

For the clone formation assay, 200 cells were seeded into 6-well culture dishes, incubated for 10 days, and then fixed and stained with crystal violet and counted. For the transient transfection, the cells were transfected with siRNA for 48 h and then suspended and seeded as described above.

### Animal experiments

All of the animal experiments were approved by the Committee on the Use and Care of Animals in Shanghai Jiaotong University. The tumor xenograft was established by injecting cells (A498-Tiki2 and A498-Con: 5 × 10^6^ cells; 769-P shRNA1 and 769-P con: 1 × 10^7^ cells) in the right flank area of 6-week-old female BALB/c nude mice (*n* = 5 per group, Shanghai Sippr-BK Laboratory Animal Co. Ltd., Shanghai, China). Tumor size was determined with calipers every 3 days. The tumor volume was calculated by the following formula: tumor volume (mm^3^) = maximum length (mm) × perpendicular width (mm^2^)/2. Before the largest tumor volume reached 1000 mm^3^, the mice were sacrificed.

### Statistical analysis

Data are shown as mean ± SD and SEM, respectively. Student's *t* test was used to compare the two groups. *P* values < 0.05 were considered significant. All statistical analyses were performed using GraphPad Prism 5 software (GraphPad Software, USA).

## SUPPLEMENTARY FIGURES


