# Supplementary material for: TIKI2 is upregulated and plays an oncogenic role in renal cell carcinoma
Source: Oncotarget. 2016 Mar 3;7(13):17212–9. doi: 10.18632/oncotarget.7873 (PMC4941381; doi:10.18632/oncotarget.7873)
Supplement: Supplementary file 1 [file oncotarget-07-17212-s001.pdf]

SUPPLEMENTARY FIGURES

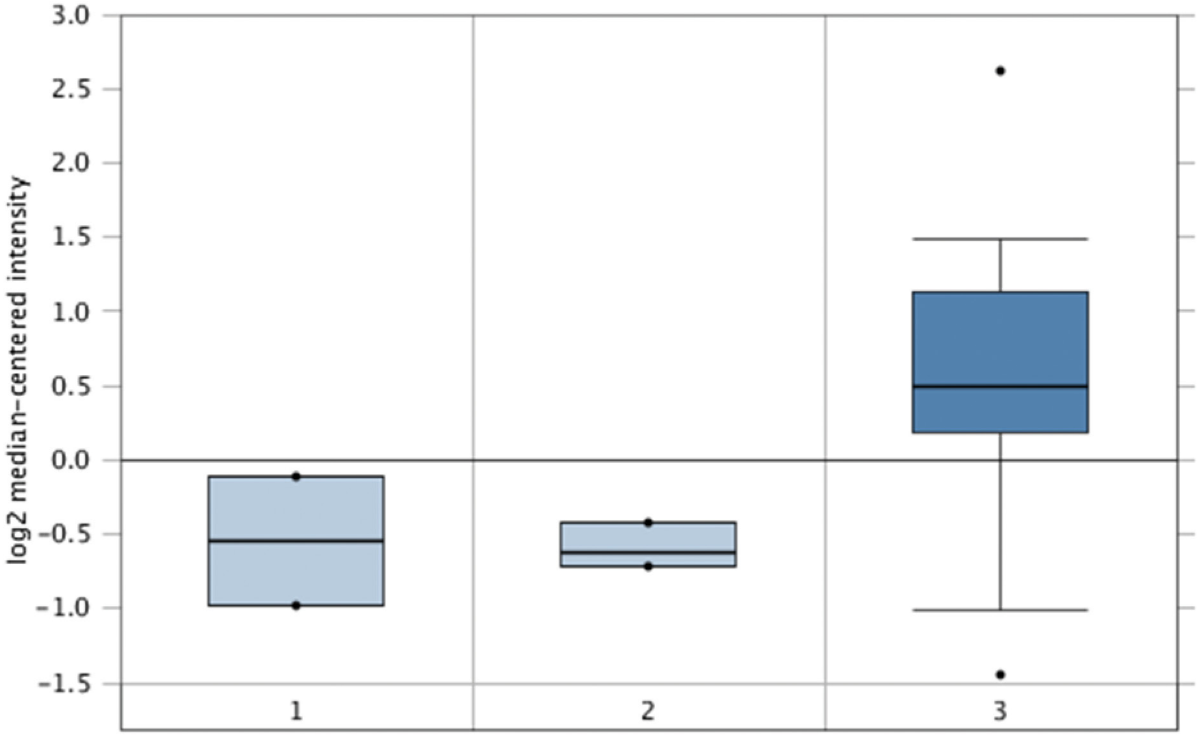

Supplementary Figure S1: TIKI2 was highly expressed in RCC. TIKI2 was highly expressed in RCC from data of Yusenko et al [27] on Oncomine. 1. Fetal kidney (n = 2). 2. Kidney (n = 3). 3. Clear Cell Renal Cell Carcinoma (n = 26). *P* = 7.14E-5.

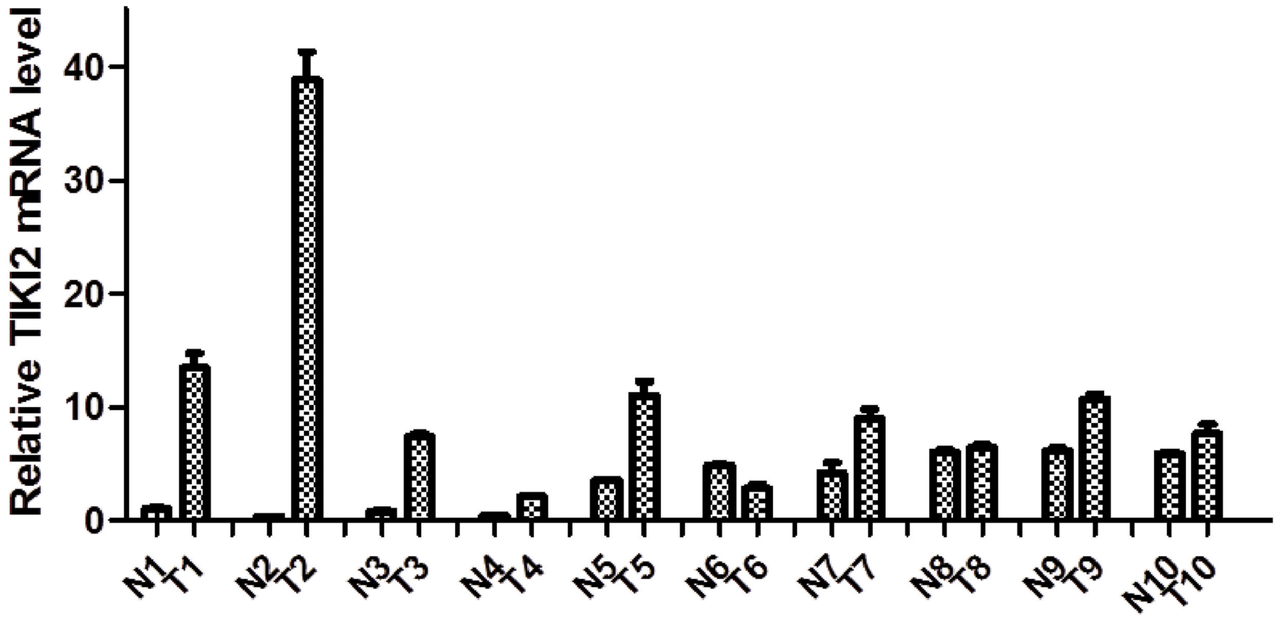

Supplementary Figure S2: TIKI2 was highly expressed in RCC specimens. TIKI2 mRNA level in each pair of RCC specimens and the corresponding non-tumor tissues were obtained using quantitative real-time PCR. Higher TIKI2 mRNA level was observed in most RCC specimens than in the corresponding non-tumor tissues (n = 10, data are mean ± SD). N: corresponding non-tumor tissues; T: RCC.

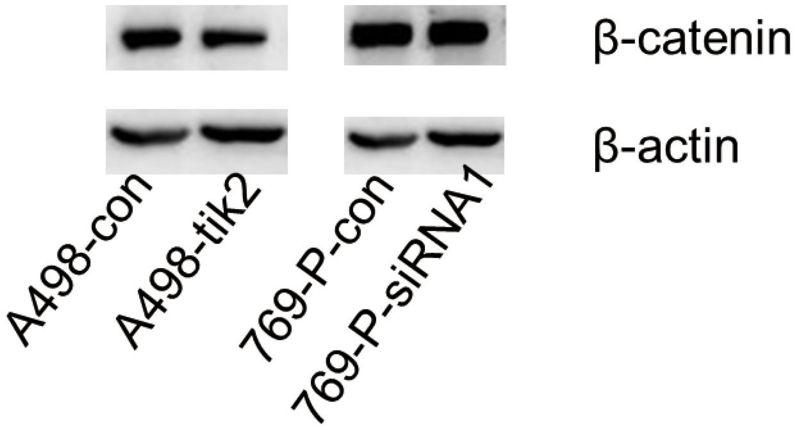

Supplementary Figure S3: TIKI2 did not affect the Wnt/β-catenin pathway in RCC cells. Expression of β-catenin in A498-Tiki2, 769-P-siRNA1 and the corresponding controls were evaluated by Western blotting. No significant differences were observed.
